# Sounds of Hidden Agents: The Development of Causal Reasoning About Musical Sounds

**DOI:** 10.1111/desc.70021

**Published:** 2025-05-02

**Authors:** Minju Kim, Adena Schachner

**Affiliations:** ^1^ Department of Psychology University of California San Diego California USA; ^2^ Teaching and Learning Commons University of California San Diego California USA

**Keywords:** animate agents, auditory cognition, causal reasoning, event reconstruction, music

## Abstract

Listening to music activates representations of movement and social agents. Why? We test whether causal reasoning plays a role, and find that from childhood, people can intuitively reason about how musical sounds were generated, inferring the events and agents that caused the sounds. In Experiment 1 (*N* = 120, pre‐registered), 6‐year‐old children and adults inferred the presence of an unobserved animate agent from hearing musical sounds, by integrating information from the sounds’ timing with knowledge of the visual context. Thus, children inferred that an agent was present when the sounds would require self‐propelled movement to produce, given the current visual context (e.g., unevenly‐timed notes, from evenly‐spaced xylophone bars). Consistent with Bayesian causal inference, this reasoning was flexible, allowing people to make inferences not only about unobserved agents, but also the structure of the visual environment in which sounds were produced (in Experiment 2, *N* = 114). Across experiments, we found evidence of developmental change: Younger children ages 4–5 years failed to integrate auditory and visual information, focusing solely on auditory features (Experiment 1) and failing to connect sounds to visual contexts that produced them (Experiment 2). Our findings support a developmental account in which before age 6, children's reasoning about the causes of musical sounds is limited by failure to integrate information from multiple modalities when engaging in causal reasoning. By age 6, children and adults integrate auditory information with other knowledge to reason about how musical sounds were generated, and thereby link musical sounds with the agents, contexts, and events that caused them.

## 1 Introduction

From infancy, music is a fundamental part of children's everyday lives (Mendoza and Fausey 2021; Politimou et al. 2018; Trehub 2003). Human *musicality*—the cognitive capacity and motivation to engage with music—is culturally universal, ancient, and early‐developing in childhood (Kim and Schachner 2023; Savage et al. 2021). This musicality involves not only sound, but also movement: Movement produces sounds, and leads to response in the form of dance (Blacking 1995; Mehr et al. 2019; Savage et al. 2015). Music also activates representations of movement in listeners, even when no movement is produced or viewed (Cannon and Patel 2021; Chen et al. 2008; Grahn and Rowe 2009). In addition, music is universally social, and even music listening activates social representations (Launay 2015; Savage et al. 2021; Steinbeis and Koelsch 2009).

What cognitive mechanisms link music with movement and the social world? Multiple mechanisms are known to play a role. Features of rhythmic sound drive the motor system via low‐level connections between auditory and motor areas (Cannon and Patel 2021; Chen et al. 2008; Cheng et al. 2022; Grahn and Rowe 2009). Some acoustic features are also perceived as analogous to specific movements, in ways that are consistent across diverse cultural contexts (Sievers et al. 2013; Mehr et al. 2018). Other perceptual attributes of sound also predict perception of animacy, movement, and emotion in music (e.g., microtiming variations: Blust et al. 2016; Juslin and Laukka 2003).

Here we test a new hypothesis. People may engage in causal reasoning about how musical sounds were generated, which may intuitively link musical sounds with the agents and events that caused them. From childhood, people reason about the causes of events they observe through a process of inference to the best explanation (Jara‐Ettinger et al. 2016; Lipton 2008; Tenenbaum et al. 2006; Xu and Kushnir 2013). This reasoning can be understood as Bayesian causal inference, operating over richly‐structured intuitive mental theories of the world (Gerstenberg and Tenenbaum 2017; Baker et al. 2017). Using Bayesian inference, adults and children also engage in *event reconstruction*, using indirect traces to infer unobserved actions an agent made in the past (Jara‐Ettinger and Schachner 2024; Lopez‐Brau et al. 2022). This includes reasoning about non‐musical sounds: From the timing of impact sounds in a pinball‐machine‐like box, adults and children over 6 years are able to infer the most likely path taken by a ball (Gerstenberg et al. 2018; Outa et al. 2022). Bayesian causal inference is thus known to occur for a broad range of non‐musical stimuli, including visually‐observed events, physical objects, and non‐musical sound sequences.

**Summary**

1. Children and adults inferred the presence of an unobserved animate agent from musical sounds’ timing; by 6‐years‐old, these inferences integrated auditory with visual information.
2. This reasoning was flexible, consistent with Bayesian causal inference: 6‐year‐olds and adults also used musical sounds’ timing to infer the structure of the visual environment.
3. Developmental change: Children ages 4 and 5 failed to integrate auditory/visual information, leading to distinct patterns of judgments across two experiments.
4. Causal reasoning and Bayesian event reconstruction provide a new cognitive mechanism by which music is linked to social cognition, with qualitative change in middle‐childhood.

Causal reasoning provides a novel cognitive mechanism by which music may activate representations of movement and social agents. If people apply this kind of reasoning to music, then based on the sounds they observe, together with some expectation about the context in which they were produced (e.g., on a xylophone), people would be able to infer the likely path of movement that caused the sounds. Based on the inferred movement, people should be able to infer whether an animate agent produced the sounds (vs. the wind, or some other inanimate force). For example, if the movement path requires moving in a self‐propelled way (jumping; changing direction), this should lead people to infer that the sounds were caused by a person (or other animate agent), as they would be impossible without an agents’ intervention (Saxe et al. 2005).

### 1.1 The Development of Causal Reasoning about Musical Sounds

Many of the underlying cognitive systems needed for this causal reasoning emerge early in life, leading to the prediction that even very young children may show causal reasoning about musical sounds. From infancy, humans have a mental theory of the physical‐mechanical world that allows them to predict and explain physical‐mechanical events, and an intuitive theory of animate agents that allows them to predict and explain social behavior (Battaglia et al. 2013; Spelke and Kinzler 2007; Liu et al. 2017, 2024). Infants expect that animate agents have unique capacities, like the ability to move in a self‐propelled way (Gelman et al. 1995; Gergely et al. 1995). As a result, when they see an event that requires self‐propelled movement (e.g., a ball emerging from behind a wall), infants infer that a hidden animate agent caused that event (Saxe et al. 2005). Young infants also link sounds with specific events, for example, expecting to hear a sound when they see an impact (Bahrick 1983, 1988; Kopp 2014; Lewkowicz 1992, 1994; Spelke 1979). By 4 years of age, children make accurate inferences about hidden causes from auditory evidence alone, using the sound of shaking a box to infer the number and nature of objects inside (Siegel et al. 2014). These findings suggest that even very young children may be able to use causal reasoning to reason about musical sounds.

However, in other contexts, children younger than 6 years fail to reason about the causes of sounds, even when adults succeed. In a pinball‐game‐like task, adults rationally inferred the path taken by the ball from the timing of sounds it made, and did so by optimally integrating visual information (other objects in the visual scene) with auditory information (Gerstenberg et al. 2018). In contrast, children younger than 6 years did not accurately infer the ball's path from the timing of sounds, and specifically failed to integrate information from vision and sound. This ability improved from 6 to 8 years of age (Outa et al. 2022). Young children also fail to make other nuanced sound source judgments before age 6 that are robust in adults, for example, failing to perceive whether water is hot or cold from the sound of it being poured (Agrawal and Schachner 2023). These findings motivate the prediction that young children may not show adult‐like causal reasoning about how sounds were generated until approximately 6 years of age (including musical and non‐musical sounds).

If young children cannot reason about the causes of sounds in an integrated way, then they may use cognitively simpler strategies when perceiving musical stimuli. For example, children may associate any and all musical sounds with animate agents, even when the particular sounds could be produced by inanimate forces (e.g., the sounds of a windchime). This association could be learned from everyday observations that musical sounds typically co‐occur with the presence of people (Launay 2015). Alternatively, young children may expect that only *orderly* musical sequences are produced by animate agents. Young children robustly expect that orderly outcomes, such as organized arrangements of objects, are produced by animate agents (Newman et al. 2010; Keil and Newman 2015; Ma and Xu 2013). Musical sequences differ in orderliness: For example, a descending scale is perceived as highly orderly, and a scrambled sequence of the same tones as less orderly (Schachner and Kim 2018). Similarly, evenly‐timed musical notes are more orderly than the same pitches with uneven, unsystematic timings between the notes. If young children expect that orderly arrangements of sounds are created by agents, then children may consistently judge that orderly musical sequences were created by animate agents—even when the particular orderly sequence could be produced by inanimate forces (e.g., a ball rolling across a tilted xylophone, producing an evenly‐timed descending scale). With age, this tendency should be reduced, as causal inference plays an increasing role. In line with this, adults in a similar task judged that orderly audio‐visual sequences were caused by hidden agents only if self‐propelled movement would be needed to produce them, and not otherwise (Schachner and Kim 2018).

### 1.2 The Current Studies

In this paper, we therefore test whether the ability to infer the causes of musical sounds develops early in life or has a protracted developmental time course. In doing so, we aim to understand the development of causal reasoning about multi‐modal stimuli (i.e., events that produce sounds, whether musical or non‐musical), and inform theories of how music becomes linked with movement and social agents (e.g., Cannon and Patel 2021; Launay 2015; Savage et al. 2021).

In a first experiment, we ask whether children aged 4–6 years, and adults, can use casual reasoning to infer whether musical sounds were produced by animate agents. We focus on reasoning about the timing of sounds, building on past evidence that older children and adults optimally infer a ball's path of movement from sounds’ timing (Gerstenberg et al. 2018; Outa et al. 2022). We ask whether children can infer whether an animate agent (vs. an inanimate object) was the cause of a sequence of musical sounds, and do so rationally, by integrating auditory information (the sounds’ timing) with visual information about the environment.

If children and adults are using Bayesian causal reasoning to infer whether an agent generated musical sounds, then their reasoning should be flexible, supporting other inferences as well (Gerstenberg et al. 2018). In a second experiment, we therefore test whether children are able to infer another aspect of the sounds’ cause: The structure of the environment in which it was produced (presented visually). Again, we test both adults and children aged 4 and 6 years, to characterize developmental change. If younger children fail to integrate information from sound with visual information, then they should fail to use sounds to reason about the structure of the visual environment.

Pre‐registrations, data, and supplementary materials for the following experiments can be found in an OSF repository at the following link: https://osf.io/bzk8x/?view_only=b90eca615105484abb2d42f26885f118.

## 2 Experiment 1

**FIGURE 1:**
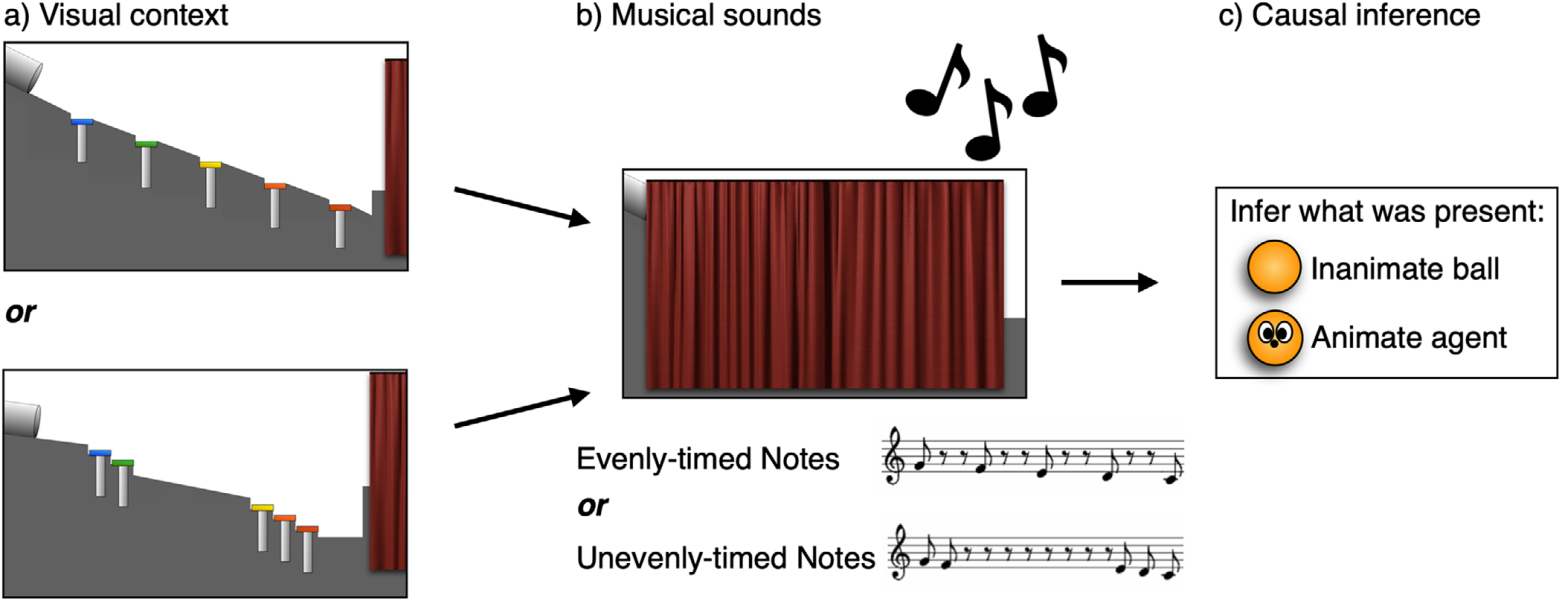
Method, Experiment 1. To test whether participants integrate auditory and visual information to infer whether an agent caused musical sounds, adults and children 4–6 years were shown visual scenes involving a xylophone (a), which was then covered with a curtain, and musical sounds occurred (b). Participants were then asked to judge whether an animate agent or an inanimate object had been present (c). Across within‐subject conditions, we manipulated the visual context in which the sounds were produced (a) and the timing of the musical sounds (b). If participants integrate information from sound and vision in causal reasoning about music, then their judgments should show an interaction between visual context and musical sounds, such that agents are judged present when the sounds would require self‐propelled motion to produce.

To test whether adults and children use causal reasoning to infer the presence of a hidden agent from musical sounds, we constructed animated stimuli in which xylophone‐like bars were embedded in a hill. On each trial, the scene was occluded by a curtain, a sound sequence was played, and participants were asked to judge whether an animate agent, or an inanimate object, had been present behind the curtain when the sounds occurred (see Figure 1).

Across conditions, we manipulated the timing of the musical sounds such that one of two sound sequences played: a descending scale with evenly‐timed notes, or a descending scale with unevenly‐timed notes. We also manipulated the visual context in which sounds were produced by changing the placement of the xylophones’ bars. Across conditions, the scene either showed a xylophone with evenly‐spaced bars or unevenly‐spaced bars (see Figure 1). The two sound sequences were timed so that, in the context of the evenly‐spaced‐bars xylophone, an inanimate ball simply rolling downhill would produce the evenly‐timed notes. In the context of the unevenly‐spaced‐bars xylophone, the ball rolling downhill would produce the unevenly‐timed notes.

If children can reason about the causes of musical sounds by integrating auditory and visual information, then when a sound sequence requires self‐propelled movement, people should infer that it was caused by an agent. In contrast, when a sound sequence could be produced by inanimate forces (a ball rolling due to gravity), this should “explain away” the agent, leading people to no longer infer that an agent is present. This pattern of reasoning—one alternative explanation weakening evidence for other explanations—is a signature of causal inference (Gopnik and Sobel 2000; Pearl 2000; Tenenbaum et al. 2006). Causal reasoning, therefore, predicts an interaction between visual context and sound sequence, such that people should infer the presence of an agent for different sound sequences in each of the two visual contexts. If children are not able to integrate visual and auditory information, then we should find no interaction. In this case, children may base their answers solely on auditory information and may expect that an agent is present when they hear more orderly sound sequences (Newman et al. 2010; Keil and Newman 2015).

### 2.1 Methods

#### 2.1.1 Participants

As pre‐registered (see OSF), *N* = 60 4‐ to 6‐year‐old children and *N* = 60 adult participants were recruited for the study. Twenty children were tested at each year of age (4 years: *M*_age_ = 54.7 months, SD = 3.57, 11 girls, 9 boys; 5 years: *M*_age_ = 66.76 months, SD = 3.34, 9 girls, 11 boys; 6 years: *M*_age_ = 77.96 months, SD = 3.41; 5 girls, 15 boys). Child participants were recruited through a database of families interested in research and participated in a 30‐min video call session via Zoom. All participating families gave informed consent and received a $5 online gift card as their reward. Three additional child participants were tested but excluded due to pre‐registered exclusion criteria: failing to finish some trials (*n* = 1) and technical issues (*n* = 2).

Adult participants were recruited from an online study pool at a large public university in Southern California (Age: *M* = 20.97 years, SD = 2.62, range = 18–31, 19 men, 41 women) and tested online. All participants gave informed consent and received credit as compensation. Seventeen additional participants were tested but excluded due to pre‐registered exclusion criteria: failing one or more attention check questions (*n* = 13) or technical issues (*n* = 4).

#### 2.1.2 Design

Using a 2 × 2 within‐subject design, each participant completed four trials. These were composed of two blocks (one for each visual context), with two trials each (one for each sound sequence). The order of the blocks and the order of test trials within a block were counterbalanced between subjects. Child participants could answer by pointing to images of an object or agent on the screen; the location of these images (left vs. right) was also counterbalanced between subjects.

#### 2.1.3 Procedure and Stimuli

Adults and children were tested with a similar procedure and stimuli, with children tested via spoken interaction over Zoom on their laptop or desktop computers and adults tested via a written task online. Animated videos and sounds were constructed using Apple Keynote, Apple GarageBand, and Apple QuickTime software. To avoid audio‐visual delays in presentation over Zoom, for children, all stimuli were presented directly in the parents’ own web‐browser, using an online platform that allowed the experimenter to control the presentation (www.slides.com). The experimenter guided parents to set up the screen with the web browser as large as possible, the experimenter in the corner, and the Zoom self‐view hidden. The experimenter then remotely controlled the presentation, while also interacting with the child over the Zoom platform.

Participants were first introduced to one of the two visual contexts (the xylophone with evenly‐spaced bars, or unevenly‐spaced bars), and viewed a familiarization video demonstrating how the bars produced sounds when struck. The visual context was described as a xylophone on a hill, or a musical staircase, where each bar makes a different note when something hits it. Familiarization videos (one for each of two visual contexts) demonstrated how the bars produced sounds: A mallet entered the scene and struck a sequence of bars before exiting the scene. The sequences in the familiarization videos were an ascending scale, played twice; once with even timing, and once with uneven timing. This was done so that the sounds heard during familiarization were equally similar to each of the two sound sequences played during the test trials. Adult participants were then asked to describe what happened and answer two attention check questions (whether each bar played the same note/different notes/animal sounds/no sounds; whether the bars were evenly‐spaced/unevenly‐spaced).

Participants were then asked to notice the pipe on the left‐hand side of the scene and told that one of two things could come out of the pipe: a ball or a cartoon character. To provide evidence of the ball's inanimacy and the character's animacy, participants viewed one brief video of each of them (showing the character moving in a self‐propelled way and the ball rolling with gravity). The two entities were also identified in ways consistent with inanimacy and animacy, specifically as a hard, heavy ball or billiard ball (for children and adults, respectively), or as a cartoon character with a name (Fred). Participants were instructed that they would be trying to figure out what came out of the pipe, but that the scene would be covered so they could not see, and would only be able to hear sounds. Child participants were prompted to practice pointing to pictures of the ball and Fred, each located on different sides of the screen.

Participants first completed two test trials in the first block, then received similar instructions for the second block (the other visual context condition), and completed the additional two test trials of the second block. During each test trial, participants watched a video in which the visual context was covered by an animated curtain, and one of two sound sequences was played: a descending scale with evenly‐timed notes (G, F, E, D, C, with fundamental frequencies of 778, 702, 647, 569, and 511 Hz), or a descending scale with unevenly‐timed notes (the same pitches, played G‐F‐E‐pause‐pause‐D‐C). Sound sequences were of equal duration (4 s). Participants were then asked to judge whether an animate agent or an inanimate object had been present when the sounds occurred, using a two‐alternative forced choice (“What came out of the pipe? Was it the ball? Or was it Fred?”). Child participants could verbally answer or respond by pointing to images of Fred/the ball. Participants then reported their certainty about this answer, again using a two‐alternative forced choice (“Was it definitely that one, or maybe that one?”). The two scales were then combined to create a single four‐point scale (1 = Definitely the ball, 2 = Maybe the ball, 3 = Maybe the agent, and 4 = Definitely the agent).

After completing all trials, adult participants were asked if they experienced any technical issues and to guess what the experiment was about before submitting their answers.

### 2.2 Results

#### 2.2.1 Adult judgments

**FIGURE 2:**
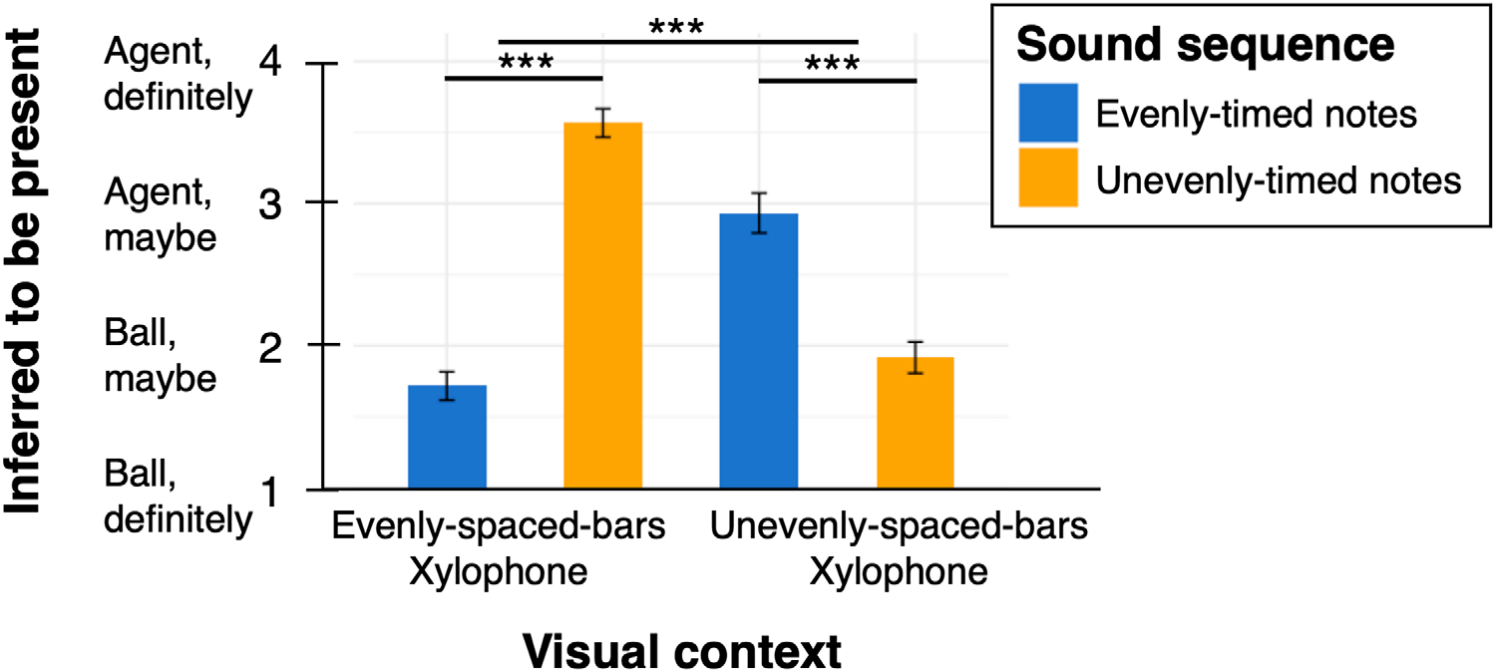
Results, Experiment 1, adult participants. In line with the use of causal reasoning and integration of information from both sound and visual context, adult participants judged that a hidden agent was more likely present (behind the curtain) when the sounds would have required self‐propelled movement to produce. Thus, in the context of a xylophone with evenly‐spaced bars, the agent was judged more likely present when unevenly‐timed notes had occurred (vs. evenly‐timed notes). In the context of a xylophone with unevenly‐spaced bars, the agent was judged more likely present when evenly‐timed notes had occurred (vs. unevenly‐timed notes). Error bars indicate ±1 SEM. *** Indicates *p* < 0.001.

As pre‐registered, to test whether participants integrated auditory information with visual information to infer the causes of musical sounds, we used an ordinal logistic regression model to predict participants’ judgments of whether the agent had been present (Definitely the ball = 1 to Definitely the agent = 4) from the predictors of visual context (evenly‐spaced‐bars/unevenly‐spaced‐bars xylophones), sound sequence (evenly‐timed/ unevenly‐timed notes), the interaction of these two factors, and participant (as a random factor). As predicted, there was a significant interaction between visual context and sound sequence in adults’ judgments of whether an agent had been present (nested model comparison, full model vs. model without the interaction term: χ^2^(1) = 112.21, *p *< 0.001, coefficient test on the interaction term: 𝛽 = –9.51, *p* < 0.001; see Figure 2). In addition, there were significant main effects of visual context (𝛽 = 6.44, *p* < 0.001) and sound sequence (𝛽 = 8.90, *p* < 0.001).

In the context of the evenly‐spaced‐bars xylophone, adult participants judged it more likely that the agent had been present after hearing the unevenly‐timed notes (*M* = 3.57, SE = 0.10) than the evenly‐timed‐notes (*M* = 1.72, SE = 0.10; Wilcoxon Test, *Z* = –6.13, *p* < 0.001). This pattern was reversed in the context of the unevenly‐spaced‐bars xylophone: In this context, participants judged it more likely that the agent had been present after hearing the evenly‐timed notes (*M* = 2.94, SE = 0.14) than the unevenly‐timed notes (*M* = 1.92, SE = 0.11; Wilcoxon Test, *Z = –3.98*, *p* < 0.001).

#### 2.2.2 Children's judgments

We analyzed children's responses using the same analysis as adults’ (ordinal logistic regression^1^), adding age (as a continuous variable) as an additional predictor. This model predicted participants’ judgments of whether the agent had been present (Definitely the ball = 1 to Definitely the agent = 4) from the predictors of visual context (evenly‐spaced‐bars xylophone or unevenly‐spaced‐bars xylophone), sound sequence (evenly‐timed notes or unevenly‐timed notes), the interaction of these two factors, age (as a continuous variable), the interaction of these three factors, and participant (as a random factor).

We found a significant three‐way interaction of age, sound sequence, and visual context on judgments of whether the agent had been present (nested model comparison of the full model vs. a smaller model without the 3‐way interaction: χ^2^(1) = 9.55, *p* = 0.002; coefficient test: 𝛽 = –0.15, *p <* 0.002), meaning that the way that sound sequence interacted with visual context changed with age. In addition, we found three significant 2‐way interactions (age*sound sequence, 𝛽 = 0.12, *p <* 0.001; sound sequence*visual context, 𝛽 = 9.72, *p <* 0.003; age*visual context, 𝛽 = 0.09, *p <* 0.007), and significant main effects of all three factors (sound sequence, 𝛽 = –8.45, *p <* 0.001; visual context, 𝛽 = –5.86, *p <* 0.01; age, 𝛽 = –0.05, *p =* 0.034).

**FIGURE 3:**
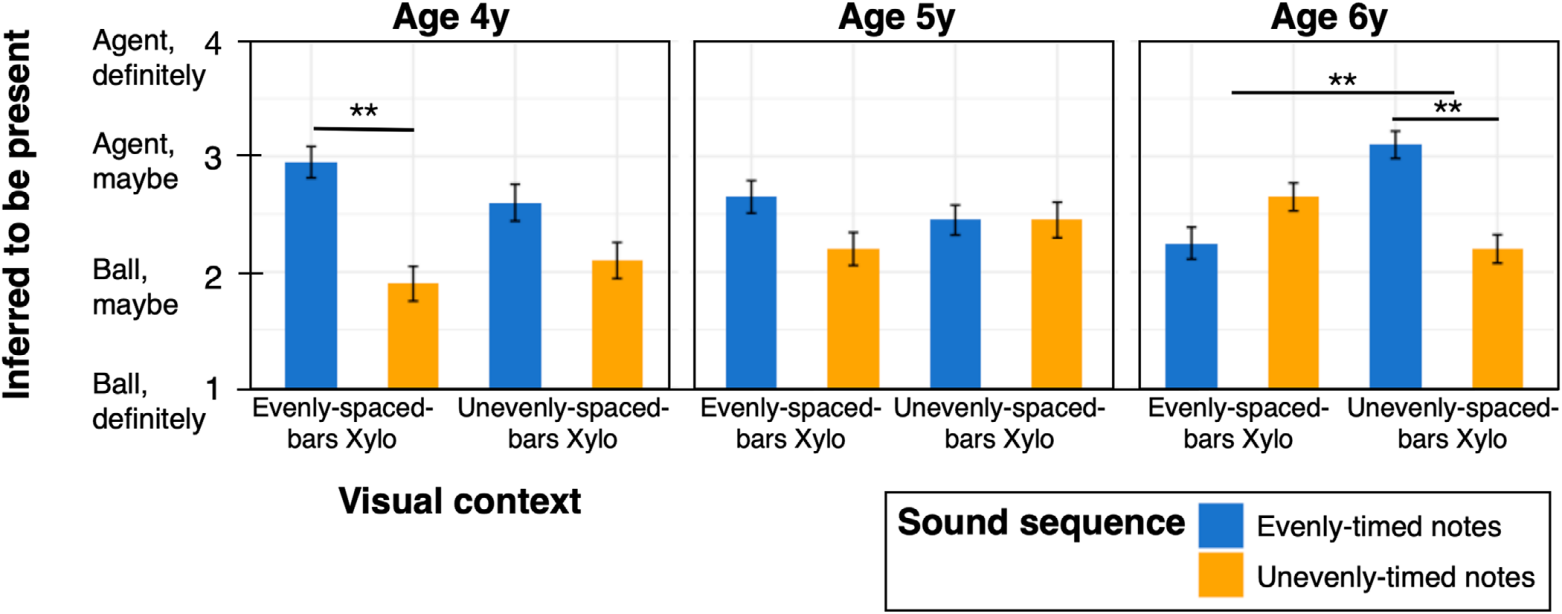
Results, Experiment 1, child participants. Like adults, 6‐year‐old children judged that a hidden agent was more likely present when the sounds would have required self‐propelled movement to produce (e.g., when evenly‐timed notes occurred in the context of a xylophone with unevenly‐spaced bars), integrating information from both musical sound and visual context. Unlike adults, 4‐year‐old children judged that a hidden agent was more likely present when evenly‐timed notes occurred, even in the context of the evenly‐spaced‐bars xylophone. Five‐year‐old children's judgments were not systematic. Error bars indicate standard errors; ** indicates *p *< 0.01.

To understand age differences and test the prediction that only 6‐year‐olds would show adult‐like reasoning, we next separately examined data at each year of age. We found that, like adults’ judgments, 6‐year‐olds’ judgments showed a significant interaction of visual context and sound sequence, in line with the use of causal reasoning (nested model comparison with vs. without interaction term: χ^2^(1) = 9.12, *p =* 0.003). In the context of the xylophone with evenly‐spaced bars, the agent was judged more likely present for unevenly‐timed notes (*M* = 2.65, SE = 0.21) than evenly‐timed notes (*M* = 2.25, SE = 0.24; Wilcoxon Test, *Z* = 122.5, *p =* 0.03). In contrast, in the context of the xylophone with unevenly‐spaced bars, the agent was judged numerically (though not statistically) more likely present for evenly‐timed notes (*M* = 3.10, SE = 0.20) than unevenly‐timed notes (*M* = 2.20, SE = 0.21; Wilcoxon Test, *Z* = 53.0, *p =* 0.26; see Figure 3).

Four‐year‐old children showed a different pattern. Their responses did not show a two‐way interaction of the visual context and the sound sequence (nested model comparison: χ^2^(1) = 0.93, *p* = 0.34), but instead a significant main effect of sound sequence (nested model comparison, χ^2^(1) = 8.97, *p =* 0.003). In both visual contexts, 4‐year‐old children judged it numerically more likely that the agent was present for evenly‐timed notes than unevenly‐timed notes (evenly‐spaced bars Xylophone: Evenly‐timed notes *M* = 2.95, SE = 0.23, Unevenly‐timed notes *M* = 1.90, SE = 0.26, Wilcoxon Test *Z* = 135.0, *p = *0.03; Unevenly‐spaced bars Xylophone: Evenly‐timed notes *M* = 2.60, SE = 0.28, Unevenly‐timed notes *M* = 2.10, SE = 0.27, Wilcoxon Test *Z =* 105.0, *p =* 0.17).

Five‐year‐olds’ judgments were not well‐predicted by the model, and showed no interaction of visual context and sound sequence (χ^2^(1) = 0.89, *p =* 0.34; nested model comparison with a model without the two‐way interaction), nor main effects (all *p*’s > 0.3; see Figure 3). We speculate that this may indicate a mix of strategies, with some 5‐year‐old children reasoning similarly to 4‐year‐olds, and others similarly to 6‐year‐olds.

### 2.3 Discussion

In Experiment 1, we found evidence that adults and 6‐year‐old children (but not younger) can integrate auditory information with visual information to infer whether musical sounds were caused by an animate agent. 4–5 year old children failed to do so, with 4‐year‐old children instead judging that an agent was present when they heard orderly, evenly‐timed sounds, regardless of the visual context.

These findings have implications regarding the scope of causal inferences that are made from sound sequences’ timing—for musical sounds, and likely non‐musical sounds as well. Previous work has shown that adults and children older than 6 years can infer a ball's path of movement from the timing of (non‐musical) impact sounds, in line with the use of Bayesian causal inference (Outa et al. 2022). Our data shows that by age 6, this system of causal reasoning also supports a further inference about the role of animate agents as causes. This inference is similarly based on the timing of impact sounds (in our stimuli, impacts on a xylophone‐like instrument). Thus, while musical sounds are tested here, causal inferences about hidden agents are likely to occur for both musical and non‐musical sound sequences (see Section 4).

These results also provide evidence that only young children (age 4) reliably treat sounds’ orderliness as a sign of animate agency. In contrast, adults and 6‐year‐old children were able to judge the more disorderly sound sequence (unevenly‐timed notes) as stronger evidence of agency when producing it required moving in a self‐propelled way (consistent with prior adult findings; Schachner and Kim 2018). This shows that as children age, their judgments about hidden agents become less dependent on perceived orderliness. Instead, by age 6, children rationally integrate multiple kinds of information (visual, auditory) to determine whether an agent was the most likely cause of an observed event, including orderly and musical sounds they hear.

## 3 Experiment 2

How flexible is the system of reasoning that supports causal inferences about musical sounds, in older children and adults? If people have a theory‐based, structured mental model of the causal process by which musical sounds are generated, then they should be capable of reasoning about more than the presence of an agent. They should also be able to flexibly infer other components of the causal system (inverted reasoning, or backward inference; Gerstenberg and Tenenbaum 2017; Jara‐Ettinger et al. 2016).

**FIGURE 4:**
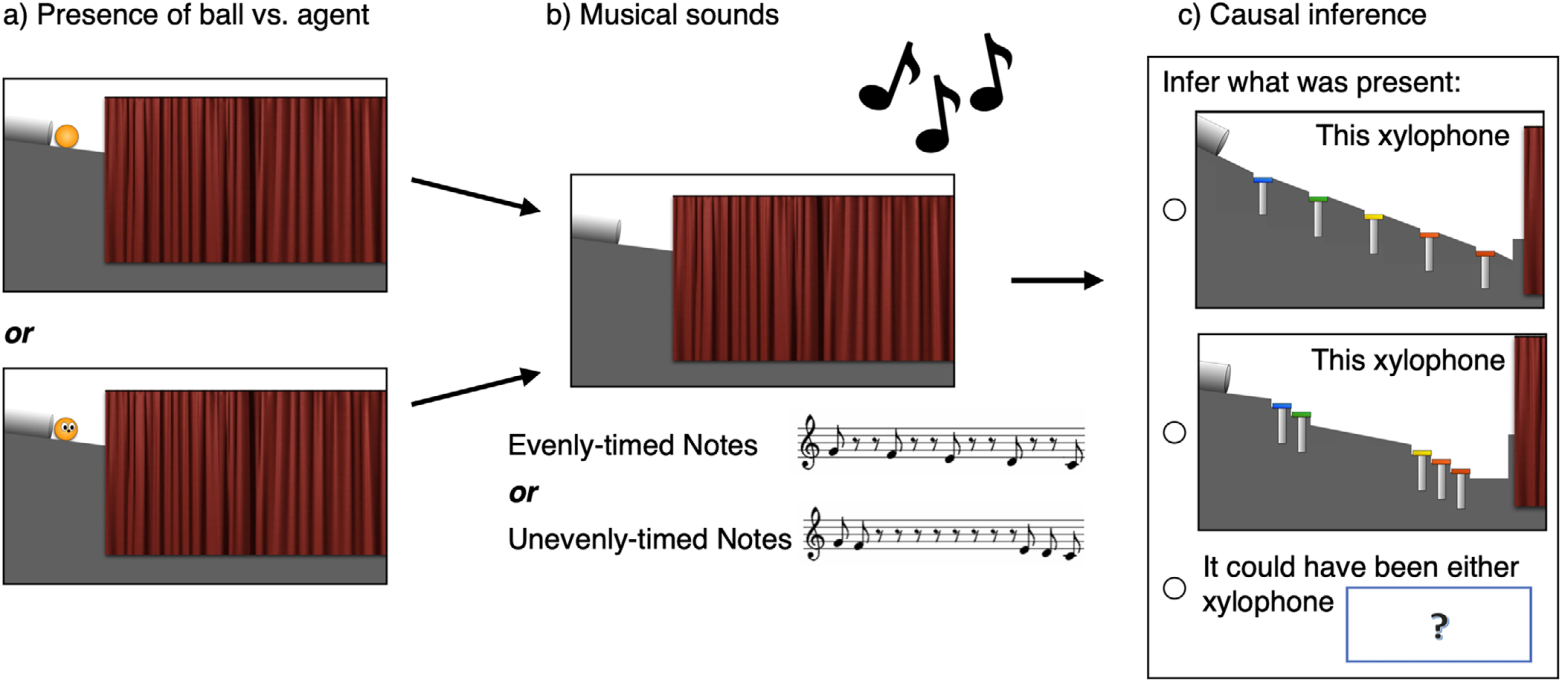
Methods, Experiment 2. To test whether participants could flexibly infer a different aspect of the causal process, in Experiment 2, adults and children ages 4 and 6 years were shown visual scenes in which the xylophone was covered by a curtain (a). Either a ball or an agent appeared and moved behind the curtain, and musical sounds occurred (b). Participants were then asked to judge which xylophone had been present, or if either xylophone could have been present (as a third option) (c). Across within‐subject conditions, we manipulated whether the ball or agent was present and the timing of the musical sounds.

In Experiment 2, we therefore switched which aspect of the situation participants were asked to infer. The visual context (i.e., the structure of the xylophone) was kept consistently covered and never revealed. However, participants were shown whether an animate agent or an inanimate object was present in the scene (at the top of the hill; see Figure 4). On each trial, participants heard a sequence of musical notes and were asked to infer which visual context was present behind the curtain. They were also given the option to indicate uncertainty (that it could be either one).

If people have a flexible, structured mental model of the causal process, then they should be able to reason about what visual context was involved in creating the musical sounds they hear. When musical sounds occur without an animate agent present (only the ball, at the top of the hill), they should be certain of the visual context, because only one context would allow the sounds to be produced through gravity alone. We therefore predict that when only the ball is present, people will select the “matching” visual context, with high confidence (e.g., evenly‐spaced bars for evenly‐timed notes). In contrast, when the agent is present, people should be less certain about which visual context is present. Because animate agents can produce many different paths of movement, they are capable of producing either sound sequence from either xylophone, making either visual context possible. When an agent is present, we therefore expect adults to express uncertainty more often, and to select the “matching” visual context less often (vs. when no agent was present). We thus measured people's judgments about which visual context was present, while giving them the option to indicate uncertainty.

We expected similar results for 6‐year‐old children. However, it is also possible that children will have a hard time recognizing their own uncertainty. Explicit metacognition is slow‐developing in childhood, and young children are notoriously overly‐optimistic about the extent of their own knowledge, underestimating their own uncertainty (Beck et al. 2011; Ghetti et al. 2013). If so, then children may select the visual context with spacing that “matches” the timing of sounds, equally in both conditions—rarely expressing uncertainty.

Last, 4‐year‐old children may not integrate visual information in their causal reasoning about sounds at all. This would be in line with findings from Experiment 1, in which 4‐year‐olds’ judgments depended solely on auditory features, disregarding the visual context. In this case, children may not see musical sounds as relevant to the question of which visual context is present, and may either express uncertainty or choose randomly between the available options. We test these predictions in Experiment 2, testing adults and children at 4 and 6 years—the two ages where clear and distinct patterns of reasoning were observed in Experiment 1.

### 3.1 Methods

#### 3.1.1 Participants

*N* = 40, 4‐ and 6‐year‐old children and *N* = 54 adult participants were recruited for the study (Adult *M*_age_ = 20.78 years, SD = 3.14, range = 18–34.0; 40 women, 14 men). Twenty children were tested at each year of age (4 years: *M*_age_ = 55.36 months, SD = 3.72, 13 girls, 7 boys; 6 years: *M*_age_ = 79.16 months, SD = 3.68; 10 girls, 10 boys). Child and adult participants were recruited in the same ways as Experiment 1, with some children additionally recruited through online advertisements on the lab's webpage. Five additional children were tested but excluded due to: failing attention check questions (*n* = 4) or having a clearly wrong birth date provided (*n* = 1). Six additional adults were tested but excluded due to failing one or more attention check questions.

#### 3.1.2 Design

Using a 2 × 2 within‐subject design, each participant completed four trials. These were composed of two blocks of trials (one for each sound sequence), with two trials each (agent vs. object present). The order of the blocks and the order of test trials within a block were counterbalanced between subjects. The order of response options of each of the two possible visual contexts (whether the evenly‐spaced xylophone or unevenly‐spaced xylophone appeared as option 1) was counterbalanced across subjects.

#### 3.1.3 Procedure and Stimuli

Adults and children were tested with a similar procedure and stimuli, with children tested via spoken interaction over Zoom and adults tested via a written task online, as in Experiment 1. Using the same video stimuli as in Experiment 1, participants were first introduced to the object or the agent (ball; cartoon character), and to the two visual contexts (evenly‐spaced xylophone; unevenly‐spaced xylophone). As in Experiment 1, adult participants were asked to describe what happened in the video, and to answer the same two attention check questions. Participants were instructed that they would see a curtain covering the scene, and would have to guess what was behind the curtain by selecting one of the two visual contexts, or indicating that it could be either one. Children were prompted to practice pointing to each of the xylophone options, and to point to the box that “means it could be either one.”

Participants then completed two test trials in the first block, and received similar instructions for the second block before completing its two additional test trials. During each test trial, participants watched a video in which the visual context was not visible, except for a small area at the top of the hill (see Figure 4); the xylophone was already covered by a curtain at the start of the video. Either the object or the agent emerged from a pipe at the top of the hill, moved down the hill, and disappeared behind the curtain. Then, one of two sound sequences occurred: either the descending scale with evenly‐timed notes, or the descending scale with unevenly‐timed‐notes (as in Experiment 1). Participants were then asked: “Which xylophone was behind the curtain?” with three answer choices: A picture of the each of the two xylophones, and a box with a question mark, with the text “[Fred/the ball] could have been on either xylophone” (see Figure 4). For child participants, the experimenter read the answer options aloud while using the mouse to point to each one (“Was it this xylophone [point]? Was it this other xylophone [point]? Or could it have been either xylophone [point]?”), and the “could have been either” option was visually placed between the two xylophone options, though it was verbally presented last (as it was for adults). Participants were then asked to explain their answer (“What made you think that?” free‐response), and children received an attention check question (“Which was in the video? Fred? Or the ball?”) before moving on to the next trial.

After completing all trials, a subset of child participants additionally engaged in a novel exploratory task, which was not part of the current experiment (children were asked to gesture the motions they believed had occurred behind the curtain). Adult participants were asked if they experienced any technical issues and to guess what the experiment was about before submitting their answers.

### 3.2 Results

#### 3.2.1 Adult judgments

**FIGURE 5:**
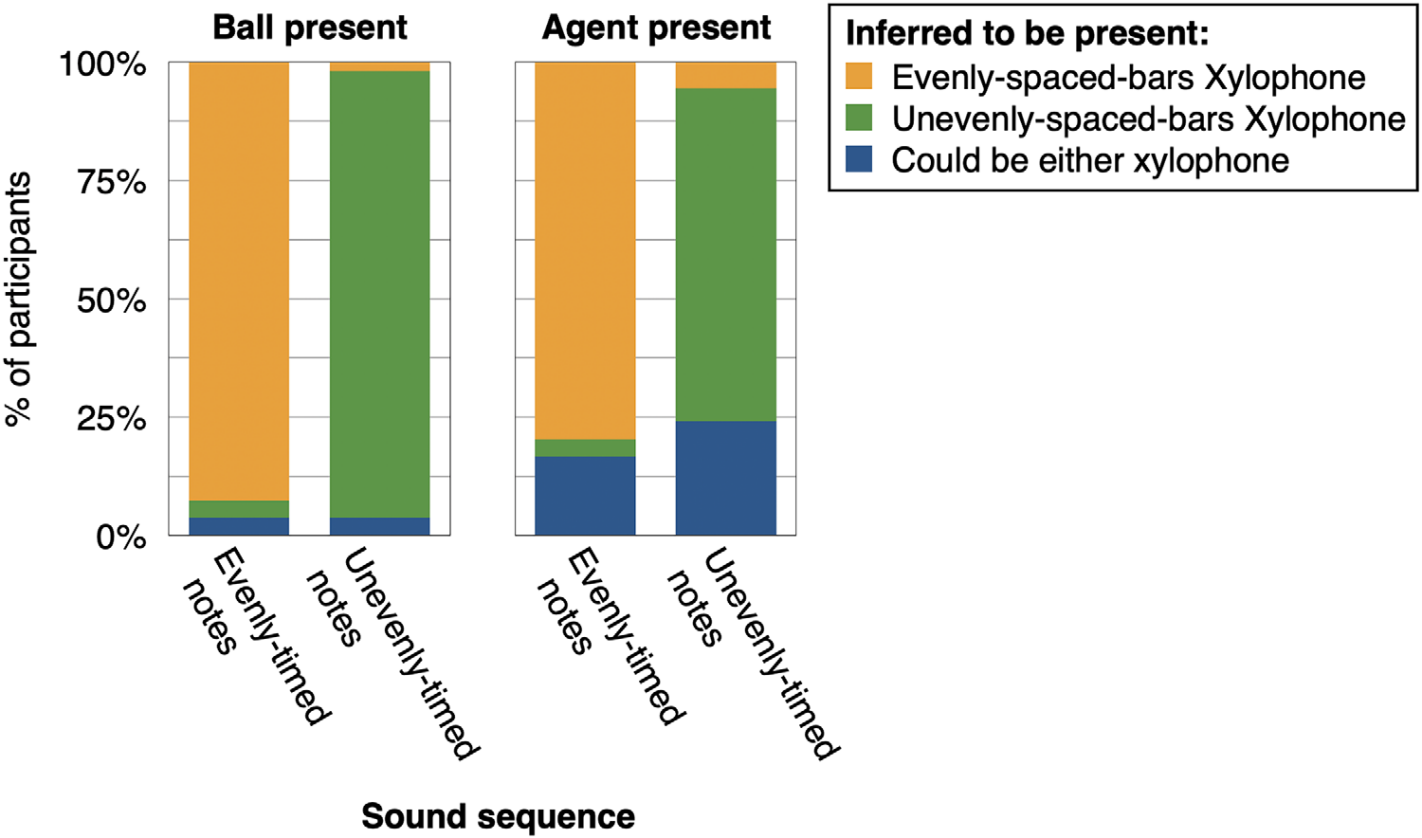
Results, Experiment 2, adult participants. Adult participants’ judgments. Adults showed flexible reasoning about multiple aspects of the causes of musical sounds, including the visual context (here). Adults frequently selected the “matching” visual context (e.g., evenly‐spaced bars for evenly‐timed notes), and more frequently did so when the ball was present versus when the agent was present. As predicted, when the agent was present, adults showed more uncertainty about the visual context, selecting “could be either” more frequently (vs. when the ball was present).

We first tested the prediction that participants would have more uncertainty about the visual context (and thus answer “it could have been either xylophone” more often) when the agent is present than when the ball is present. As predicted, we found that participants chose “could have been either xylophone” (vs. any other option) more often when the agent was present, than when the ball was present (20.4% vs. 3.7% of trials; McNemar's test, *χ^2^* (1) = 52.07, *p* < 0.0001). This effect held both on trials where the evenly‐timed notes occurred (16.7% vs. 3.7% of trials; *χ^2^* (1) = 28.90, *p* < 0.0001), and on trials where the unevenly‐timed notes occurred (24.1% vs. 3.7% of trials; *χ^2^* (1) = 22.20, *p* < 0.0001, see Figure 5).

We next tested the prediction that participants should most frequently select the “matching” visual context (e.g., evenly‐spaced bars for evenly‐timed notes), and should do so more often when the ball (vs. the agent) was present. As predicted, on trials where the ball was present, participants chose the matching visual context on the vast majority of trials (93.5% of trials). When the agent was present, they chose the matching context significantly less often, though still at a high rate (75.0% of trials, vs. 93.5% of trials, *χ^2^* (1) = 60.56, *p* < 0.0001) (See Figure 5).

#### 3.2.2 Children's judgments

**FIGURE 6:**
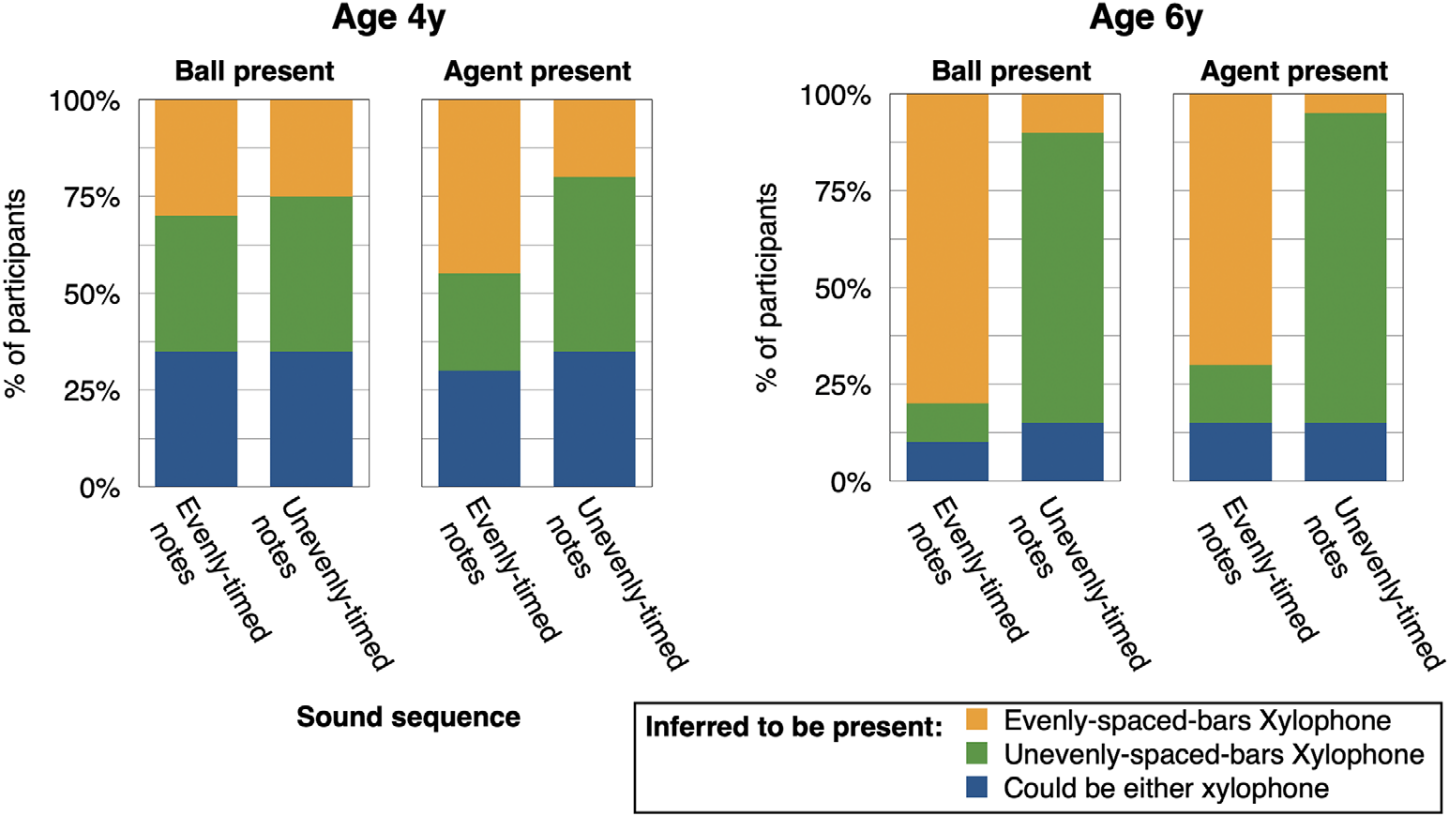
Results, Experiment 2, child participants. Children's judgments. Like adults, 6‐year‐old children frequently selected the “matching” visual context (e.g., evenly‐spaced bars for evenly‐timed notes). Unlike adults, 6‐year‐olds did not show more uncertainty in the agent condition than the ball condition (i.e., they did not select the “could be either xylophone” option at a higher rate). 4‐year‐old children selected each of the three possible responses at equivalent rates, which did not differ from chance.

In examining children's responses, we first found that like adults, 6‐year‐old children most frequently selected the matching visual context (76.3% of trials; vs. 10% for the mismatching context; and 13.8% for “could be either”; Chi‐squared test vs. 33.33% chance, χ^2^(2) = 66.48, *p* < 0.0001; see Figure 6). In contrast to adults, however, 6‐year‐olds chose the matching visual context equally often when the agent was present versus when only the ball was present (75.0% vs. 77.5% of trials; McNemar's test *χ^2^* (1) = 0.00, *p* = 1.00). 4‐year‐olds selected each of the three possible responses at equivalent rates, which did not differ from chance (*χ^2^* (2) = 0.48, *p =* 0.79; Chi‐squared test comparing the proportion of each response to a 33.33% chance).

### 3.3 Discussion

In Experiment 2, we find evidence that adults have a flexible, structured mental model of the causal process by which musical sounds are generated. When reasoning about musical sounds, adults were not limited to inferring the presence of agents (as in Experiment 1); they were able to invert this reasoning process and infer which visual context must have been present.

When musical sounds occurred *without* an agent present, and only a ball (at the top of the hill), adults reliably selected the “matching” visual context (e.g., evenly‐spaced bars for evenly‐timed notes), and only rarely expressed uncertainty. In contrast, when the agent was present, adults expressed uncertainty more often, and selected the “matching” visual context less often. This provides evidence that adults are reasoning about the causal process, and recognize that the agent is capable of producing either musical sequence from either xylophone. These findings, together with Experiment 1, provide evidence that adults have a causal mental model of how musical sounds are generated, which is flexible and structured.

Six‐year‐old children also showed evidence of causal reasoning: Like adults, they systematically picked the xylophone that matched the sounds (e.g., unevenly‐spaced bars for unequally‐timed notes). However, unlike adults, they did not express uncertainty more frequently when the agent was present in the scene, versus when the ball was present. We hypothesize that children's immature metacognition likely plays a role in this developmental difference. In many explicit tasks, 6‐year‐old children are less meta‐aware of their uncertainty as compared to adults (Beck et al. 2011; Ghetti et al. 2013). This may explain why 6‐year‐old children did not express uncertainty more often in the agent condition, and is consistent with 6‐year‐olds’ engaging in otherwise adult‐like causal reasoning about the sounds (e.g., in Experiment 1).

Interestingly, when the animate agent was present, even some adult participants continued choosing the visual context that matched the sound. This pattern of judgments can also be understood as rational: People intuitively expect that other agents act to minimize effort while maximizing reward (Jara‐Ettinger et al. 2016). In our task, participants do not have any reason to believe that one sequence of tones is more rewarding than the other. They may, therefore, expect the agent to take the least effortful path, directly downhill—rather than making additional effort to produce a different, more difficult melody. This may explain why both 6‐year‐old children and adults often selected the matching visual context when the agent was present.

Four‐year‐old children did not systematically pick any answer, instead choosing between the three answers at a rate no different from chance. This suggests that 4‐year‐old children did not consider musical sounds to be relevant or provide information about the visual context, even when they show physical objects that apparently produce sound. This is consistent with the idea that 4‐year‐old children do not integrate visual information into their reasoning about musical sounds, and may not engage in integrated causal reasoning about musical sounds.

## 4 General Discussion

Music is deeply linked to the social world, yet the cognitive and developmental bases of this connection are not fully understood (Savage et al. 2021; Steinbeis and Koelsch 2009). Here we find that from childhood, causal reasoning provides a novel cognitive mechanism by which sounds activate representations of social agents. Thus, 6‐year‐old children and adults inferred the presence of an unobserved animate agent from hearing musical sounds, by integrating information from the sounds’ timing with knowledge of the visual context (Experiment 1). Consistent with the use of Bayesian causal inference, this reasoning was flexible, allowing people to make inferences not only about unobserved agents, but also about the structure of the visual environment in which sounds were produced (Experiment 2). Last, we found evidence of developmental change: Younger children failed to integrate auditory and visual information, focusing solely on auditory features (Experiment 1), and failing to connect sounds to visual contexts that produced them (Experiment 2).

These findings show that from early school age, people can use sounds’ features to reason about unobserved events that caused them. The flexible and integrated nature of this reasoning suggests it may involve *event reconstruction*, a form of Bayesian inference by which people infer the particular past events and actions that caused things they observe (Lopez‐Brau et al. 2022). From childhood, people use event reconstruction to reason about objects’ history, inferring how past events and social agents shaped objects’ physical features (Jara‐Ettinger and Schachner 2024; Gweon et al. 2017; Pelz et al. 2020). The current work suggests that event reconstruction likely extends beyond physical objects and beyond visual input to also play a role in auditory cognition. This form of reasoning provides a parsimonious explanation for how even recorded, instrumental music—lacking human voices, and far removed from the events that originally created it—can activate representations of movement and people (Sievers et al. 2013; Steinbeis and Koelsch 2009).

While our stimuli took the form of musical sounds, there is reason to believe that this type of reasoning likely generalizes to non‐musical sounds as well. Adults and children over 6 years old use Bayesian causal inference to reason about the physical‐mechanical causes of non‐musical sounds, for example, to infer a ball's path from the timing of its impact sounds (Gerstenberg et al. 2018; Outa et al. 2022). In the current work, adults and children used the timing of impact sounds—in particular, impacts on a xylophone‐like instrument—to make the further inference that the sounds were caused by an animate agent. Since non‐musical sounds also include impact sounds, it is likely that people use causal inference to infer hidden agents in a similar way across both musical and non‐musical sound sequences. Thus, more broadly, our work provides evidence that people rationally infer not only the physical‐mechanical causes of sounds they hear, but social causes as well.

The current studies particularly focus on the use of timing cues. Causal reasoning about sound likely also pulls in information from other acoustic features, such as timbre, pitch, and loudness (which hold information about the source of the sound and what movements are being produced). When reasoning about non‐musical sounds, people are known to use a wide variety of nuanced acoustic features to make sound source attributions (Agrawal and Schachner 2023; Cusimano et al. 2018; Lutfi et al. 2008; Traer et al. 2021); this is likely the case for musical sounds as well. There may be individual differences regarding which acoustic cues are used or emphasized: From childhood, people differ in their ability to perceive and remember pitch and rhythm (Gingras et al. 2015; Gordon et al. 2015). These individual differences in acoustic perception may impact people's ability to optimally infer the causes of sounds, for example, by failing to perceive information from pitch, in the case of congenital amusia (Peretz and Vuvan 2017). Developmental changes in auditory sensitivity may also impact judgments by changing which frequencies are audible (Jensen and Neff 1993; Maxon and Hochberg 1982; Saffran et al. 2006). Future experiments should test the extent to which adults and children use features like pitch, timbre, or loudness to reason about how sounds were generated. This could be tested for both musical and non‐musical sounds, extending current methods and those of prior work (Outa et al. 2022) to manipulate and test the role of different acoustic features in causal reasoning, and explore how their relative salience changes or remains stable over development.

### 4.1 Characterizing Developmental Change

What explains younger children's failures and the developmental change in children's reasoning between 4 and 6 years of age? These failures may reflect limitations in young children's ability to integrate auditory and visual information when engaging in causal reasoning. In previous work, children younger than 6 also failed to integrate information from vision and sound during causal reasoning about non‐musical impact sounds (Outa et al. 2022), and failed to make other nuanced sound source judgments on which adults and older children reliably succeeded (hearing water temperature; Agrawal and Schachner 2023).

Young children's failure to integrate multimodal information may initially appear surprising: When perceiving multimodal events, even infants are able to integrate audio‐visual information, for example, to form a single coherent percept of a multimodal event (Bahrick 1988; Hannon et al. 2017; Kopp 2014; Lewkowicz 1994; Spelke 1979). However, the current tasks require children to integrate auditory and visual information as part of explicit causal reasoning, rather than as part of low‐level perceptual processing. Such explicit causal reasoning is likely to develop on a different trajectory from perceptual or implicit aspects of multimodal integration.

In line with our findings of developmental change, the ability to rationally integrate information from multiple sensory modalities is known to develop in a protracted manner from 6 to 12 years of age, with younger children showing notable failures to integrate multimodal input across multiple tasks requiring explicit judgments (Barutchu et al. 2009; Gori et al. 2008; Nardini et al. 2008). For example, in a task where children were asked to judge which of each pair of physical blocks was taller, young children used either visual or haptic information, but not both (Gori et al. 2008). Similarly, when reasoning about the causes of musical sounds, 4‐year‐old children in our tasks appeared to focus solely on auditory information, and thus failed to integrate visual information (in Experiment 1), and were unable to infer the visual context (in Experiment 2). The observed developmental change from 4 to 6 years may thus reflect early difficulty (and improvement with age) in integrating information from multiple sensory modalities when engaging in explicit causal inference.

Four‐year‐old children's judgments may also be seen as reflecting an even broader tendency Piaget termed centration (Piaget and Inhelder 1969). Across a variety of tasks, children ages 2–6 years often focus on a single salient aspect of a situation, while failing to consider other relevant aspects (e.g., using the height of a column of liquid to judge its quantity, while ignoring its width; Piaget and Inhelder 1969). In line with this tendency, 4‐year‐old children in our experiments used information from only one modality (sound), while failing to consider other aspects of the situation (visual context).

Young children (though not older children) may particularly rely on the perceived orderliness of the sound sequences in their reasoning. Infants and young children show an early‐emerging bias to link orderliness with animate agents, attributing orderly outcomes to the actions of agents (Newman et al. 2010; Keil and Newman 2015; Ma and Xu 2013). Four‐year‐olds’ distinct, systematic pattern of judgments in Experiment 1 are in line with this: These children judged that an agent was more likely present when they heard evenly‐timed notes (vs. unevenly‐timed notes), thus attributing agency when they perceived orderly sounds. Results of Experiment 2 are also consistent with this: A tendency to link agents with order would not support further inferences about the visual context, which 4‐year‐olds failed to make. Notably, we find that from 4 to 6 years, children's judgments about hidden agents become less dependent on perceived orderliness (consistent with prior adult findings; Schachner and Kim 2018). Instead, adults and older children rationally integrated multiple kinds of information (visual, auditory) to infer whether an agent was the most likely cause of observed sound sequences, doing so similarly whether the sounds were orderly or disorderly.

Overall, our findings support a developmental account in which, before age 6, causal reasoning about music is limited by failure to integrate information from multiple modalities in causal reasoning. In the absence of this integration, children's judgments appear to be driven by the detection of particular auditory features, like orderliness, and are not flexible, but limited to specific, narrow judgments such as the presence of agents.

Might younger children ever reason about the causes of musical sounds? In several studies, infants have been found to engage in implicit forms of reasoning even when older children fail analogous explicit tasks (Keen 2003; Wynn 1992). It is therefore possible that infants may show implicit reasoning about the causes of the musical sounds well before they succeed at explicit reasoning. Infants show much of the needed conceptual understanding via implicit tasks, including expecting only animate agents to produce self‐propelled movement (Spelke and Kinzler 2007; Gelman et al. 1995; Gergely et al. 1995), inferring that a hidden agent must be the cause of events that require self‐propelled movement (Saxe et al. 2005), and expecting to hear a sound when they see an impact (Bahrick 1983, 1988; Kopp 2014; Lewkowicz 1992, 1994; Spelke 1979). However, like 4‐year‐olds in the current work, infants may also use music's orderly nature to attribute agency, since infants typically link orderly outcomes with agents (Keil and Newman 2015). Future work using looking time methods should investigate whether and how infants implicitly reason about agents as causes of musical sounds. If infants infer the presence of an agent from hearing musical sounds, this could help explain infants’ interest in instrumental music (Kragness et al. 2023; Saffran et al. 2000), as infants are known to preferentially attend to social stimuli (Frank et al. 2009; Johnson et al. 1991; Simion et al. 2008).

### 4.2 Generalizing to Everyday Music Listening

The current data show that people are capable of causal reasoning about musical sounds, when prompted. Does this process occur spontaneously during everyday music listening? We hypothesize that in real‐world music listening, causal reasoning may occur when deeply engaging with music, as in active or creative music listening (Dunn 1997; Kratus 2017; Peterson 2006), or during mental imagery or creation of mental narratives related to musical content (Margulis et al. 2019).

Our stimuli differ from typical modern music listening in that listeners were able to see the contexts in which the sound was produced (i.e., the xylophones). While this is typical for live performances, modern Western children and adults more often hear recorded music, without seeing the scene where it is produced (Krause et al. 2015; Mendoza and Fausey 2021). Bayesian causal reasoning should easily generalize to this situation. In particular, this would introduce a greater amount of uncertainty to the causal reasoning process, leading people to rely more strongly on their prior knowledge of how sounds are typically created, to make inferences. For example, a person may use the sounds’ timbre to infer that it is a musical instrument, and narrow down the identity of the instrument (e.g., a xylophone or piano). They may also use prior experience to expect that the bars or keys are placed in ascending order, while also having more uncertainty about the instrument's type, structure, and size. People should be able to reason about this more uncertain situation by weighing the likelihood of the musical sequence under a variety of hypothesized causal events and then marginalizing over those possibilities (Griffiths and Tenenbaum 2009).

Naturalistic music is also typically longer than the musical sequences in our stimuli. Longer musical sequences likely provide even stronger evidence that an agent must be the cause, since producing long musical sequences almost always requires changing direction and speed in a self‐propelled and intentional way, in almost any plausible physical environment. We thus expect that our findings generalize to real‐world music listening, even with greater uncertainty about context and longer musical sequences.

### 4.3 Individual Differences, and the Role of First‐Person Experience

There may also be individual differences in the tendency to engage in causal reasoning about music. In part, this may be driven by differences in first‐person experience: First‐person active experience with music production may lead musicians to more fully represent and simulate the actions involved in music production during music listening (Meister et al. 2004), which may support causal reasoning through event reconstruction.

First‐person active experience may play a role in children's causal reasoning about music, as well. First‐person experience is known to scaffold children's conceptual development. For example, giving young infants earlier‐than‐usual first‐person experience with reaching for objects (using Velcro “sticky mittens”) impacts their action understanding, allowing them to understand that others’ actions are goal‐directed (van den Berg and Gredebäck 2021). First‐person experience also impacts perception of multisensory musical stimuli: 6‐month‐old infants given experience using a drum (and not those who merely observed drumming) were subsequently more sensitive to multisensory synchrony in others’ drumming actions (Gerson et al. 2015). Relevant first‐person experience may also scaffold children's causal reasoning about how music is generated, by helping them understand how movements and physical objects relate to sound production. Future work could test this by giving 4‐ and 5‐year‐old children a first‐person experience interacting with a xylophone (e.g., playing it; observing how a ball rolling across it also produces sound). By hypothesis, this experience could impact children's understanding of the causal process by which sounds are produced, supporting earlier use of integrated, rational inference to posit unobserved movements and hidden agents causing the sounds.

## Author Contributions

**Minju Kim**: investigation, writing — original draft, writing ‐ review and editing, visualization, formal analysis, data curation, methodology, conceptualization. **Adena Schachner**: conceptualization, writing — review and editing, project administration, supervision, methodology, visualization, funding acquisition, resources.

## Conflicts of Interest

We have no known conflicts of interest.

## Data Availability

The data that support the findings of this study are openly available in an OSF repository https://osf.io/bzk8x/?view_only=b90eca615105484abb2d42f26885f118.

## Endnotes

1 In our pre‐registration, we planned analyses that treated the dependent variable as continuous, rather than ordinal. Here we deviate from this plan to report the correct analysis type (ordinal logistic regression). Results from both kinds of analyses are consistent with one another (preregistered repeated‐measures ANOVA also finds the significant 3‐way interaction, *p* = 0.007; three significant 2‐way interactions, age*sound sequence, *p* = 0.003; sound sequence*visual context, *p* = 0.009; age*visual context, *p =* 0.014; and significant main effects of sound sequence, *p* = 0.001; visual context, *p* = 0.02; and age group, *p* = 0.04).
